# Cyclic stretch regulates immune responses via tank‐binding kinase 1 expression in macrophages

**DOI:** 10.1002/2211-5463.13526

**Published:** 2022-12-01

**Authors:** Anna Nakagawa, Sumio Hayakawa, Yinglan Cheng, Azusa Honda, Ryo Yuzawa, Rei Ogawa, Yumiko Oishi

**Affiliations:** ^1^ Department of Biochemistry and Molecular Biology, Graduate School of Medicine Nippon Medical School Tokyo Japan; ^2^ Department of Plastic, Reconstructive and Aesthetic Surgery Nippon Medical School Tokyo Japan

**Keywords:** cyclic stretch, inflammatory cytokines, macrophage, NF‐κB phosphorylation, tank‐binding kinase

## Abstract

Macrophages distributed in tissues throughout the body contribute to homeostasis. In the inflammatory state, macrophages undergo mechanical stress that regulates the signal transduction of immune responses and various cellular functions. However, the effects of the inflammatory response on macrophages under physiological cyclic stretch are unclear. We found that physiological cyclic stretch suppresses inflammatory cytokine expression in macrophages by regulating NF‐κB activity. NF‐κB phosphorylation at Ser536 in macrophages was inhibited, suggesting that tank‐binding kinase (TBK1) regulates NF‐κB activity during physiological stress. Moreover, TBK1 expression was suppressed by physiological stretch, and TBK1 knockdown by siRNA induced the suppression of NF‐κB phosphorylation at Ser536. In conclusion, physiological stretch triggers suppression of a TBK1‐dependent excessive inflammatory response, which may be necessary to maintain tissue homeostasis.

## Graphical abstract

Macrophages distributed in tissues throughout the body contribute to homeostasis. We found that physiological cyclic stretch suppresses inflammatory cytokine expression by regulating NF‐κB activity. Moreover, TBK1, which is an adaptor molecule linking receptors to its downstream gene expression, was also suppressed. Physiological stretch triggers suppression of a TBK1‐dependent excessive inflammatory response, which may be necessary to maintain tissue homeostasis.

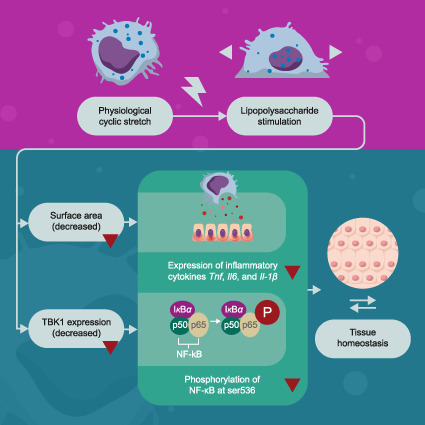

**Abbreviations**
DAMPs: damage‐associated molecular patterns
IFN: interferon
IL: interleukin
IRF: interferon regulatory factor
LPS: lipopolysaccharide
PAMPs: pathogen‐associated molecular patterns
TBK: tank‐binding kinase
TLRs: Toll‐like receptors
TNF: tumor necrosis factor

Macrophages are hematopoietic cells of the myeloid lineage that are specialized in phagocytosis and respond to diverse environmental signals. They actively maintain the steady state by secreting and responding to cytokines and chemokines [1]. Recent studies have reported that inflammatory and physiological movement responses are closely related [2, 3].

The immune system, responsible for inflammatory responses, can be broadly classified into two systems: innate and adaptive systems. The innate immune system works at the front line of host defense [4, 5], and is rapidly activated not only by pathogen‐associated molecular patterns (PAMPs), which are the components of bacteria and viruses, but also by damage‐associated molecular patterns (DAMPs), which are released as endogenous danger signals. Toll‐like receptors (TLRs) and other sensor molecules involved in the activation of the innate immune system recognize foreign substances such as PAMPs or DAMPs, and rapidly secrete pro‐inflammatory cytokines such as interleukin (IL)‐6, tumor necrosis factor (TNF), IL‐1b, and antiviral cytokines such as interferons (IFNs). TLR4 is a key receptor for the detection of lipopolysaccharides (LPS) and some saturated fatty acids, and plays an important role in triggering the host defense response. Ligand binding induces receptor dimerization, which facilitates the recruitment of other signal transducers into complexes such as myddosome, and its response activates the inflammatory response via NF‐κB. Downstream TLR4 signaling not only activates NF‐κB but also triggers interferon regulatory factor 3 (IRF3) as an antiviral response via tank‐binding kinase (TBK1). The activation of NF‐κB and IRF3 induces the production of pro‐inflammatory cytokines and type I interferon [4].

Physiological movement is necessary to maintain tissue homeostasis. Mechanical cyclic stretch as a physiological movement is detected by mechanoreceptors on the cell surface, which enables the conversion of external mechanical stimuli to biochemical signals in the cell, activating downstream signaling pathways [6, 7, 8]. This activation varies depending on whether the cell is exposed to physiological stretch stress. The restricting physiological movement might disrupt homeostasis at cellular and tissue levels. In other words, the restriction of movement associated with injury or aging may increase the risk of dysregulation of the inflammatory response, including the innate immune system, leading to a persistent inflammatory response such as chronic inflammation. However, the detailed mechanisms underlying the coordination of physiological movement and inflammatory responses at the molecular level remain unclear.

Here, morphological changes were measured after LPS stimulation of macrophages with 5% mechanical cyclic stretch conditions, reported as physiological stretch [9, 10]. Quantitative polymerase chain reaction (qPCR) analysis was performed to detect the changing inflammatory cytokine levels in macrophages in response to cyclic stretching and LPS stimulation. Our findings support the importance of physiological mechanical stress, such as movement, in the innate immune response as a part of host defense.

## Materials and methods

### Reagents and antibodies

LPS (*Escherichia coli* O55:B5) was purchased from Sigma‐Aldrich (St. Louis, MO, USA). SB203580, Gö6983, and LY294002 were purchased from Calbiochem (Merck KGaA, Darmstadt, Germany). The following antibodies were used: anti‐GAPDH (Abcam, Cambridge, UK), anti‐NF‐κB p65 (Santa Cruz Biotechnology, Dallas, TX, USA), anti‐phospho‐NF‐κB p65 (Ser536) (Cell Signaling Technology, Danvers, MA, USA), anti‐TBK1 (Cell Signaling Technology), and anti‐TLR4 (Santa Cruz Biotechnology).

### Cell culture

RAW264.7 macrophages were cultured in Dulbecco's Modified Eagle Medium (Nacalai Tesque Inc., Kyoto, Japan) supplemented with 10% heat‐inactivated fetal bovine serum (Hyclone, GE Healthcare, Logan, UT, USA) and penicillin–streptomycin (Nacalai Tesque Inc.). RAW264.7 macrophages (5 × 10^5^ cells per well) were seeded into a stretch chamber and then cultured for 4 h in a culture medium. The cells were then exposed to 5% cyclic stretch (1 count·min^−1^) for 16 h using the Shell Pa cell stretching system (Menicon Life Science, Nagoya, Japan) at 37 °C under 5% CO_2_, followed by stimulation with 100 ng·mL^−1^ LPS under 5% stretch conditions.

### RNA‐mediated interference

Chemically synthesized siRNA (Mm_Tbk1_4992) and control siRNA (siRNA negative control) were obtained from Sigma‐Aldrich. Cells were transfected with 50 nm siRNA using Lipofectamine RNAiMAX (Invitrogen, Carlsbad, CA, USA) and incubated for 48 h, followed by stimulation with 100 ng·mL^−1^ LPS for 4 h.

### RNA isolation, reverse transcription, and quantification of gene expression

Total RNA was isolated from cultured cells using ISOGEN (Nippon Gene Co. Ltd., Toyama, Japan), according to the manufacturer's protocols. Complementary DNA was synthesized from 0.5 μg total RNA using ReverTra Ace qPCR RT master mix with gDNA remover (Toyobo, Osaka, Japan). A KAPA SYBR fast qPCR kit (Nippon Genetics Co., Ltd.) and the StepOnePlus Real‐Time PCR system (Applied Biosystems, Foster City, CA, USA) were used for qPCR, according to the manufacturer's instructions. The thermal cycling was performed at 95 °C for 20 s, followed by 40 cycles at 95 °C for 3 s and 60 °C for 30 s. Target gene expression was normalized to that of GAPDH in RAW264.7 macrophages. The following primers were used: *Il6*‐F 5′‐ATGGATGCTACCAAACTGGAT, *Il6*‐R 5′‐TGAAGGACTCTGGCTTTGTCT, *Tnf*‐F 5′‐CAGGCGGTGCCTATGTCTC, *Tnf* ‐R 5′‐CGATCACCCCGAAGTTCAGTAG, *Il1b*‐F 5′‐TGGGCCTCAAAGGAAAGAAT, *Il1b*‐R 5′‐CAGGCTTGTGCTCTGCTTGT, *Tlr4*‐F 5′‐ ATGGCATGGCTTACACCACC, *Tlr4*‐R 5′‐GAGGCCAATTTTGTCTCCACA, *Ifnb*‐F 5′‐CAGCTCCAAGAAAGGACGAAC, *Ifnb*‐R 5′‐GGSAGTGTAACTCTTCTGCAT, *Tbk1*‐F 5′‐ACTGGTGATCTCTATGCTGTCA, *Tbk1*‐R 5′‐TTCTGGAAGTCCATACGCATTG, *Gapdh*‐F 5′‐AATGTGTCCGTCGTGGATCT, and *Gapdh*‐R 5′‐CATCGAAGGTGGAAGAGTGG.

### Immunoblotting

Whole‐cell extracts were obtained by lysing cells in a buffer containing 50 mm Tris–HCl (pH 7.6), 250 mm NaCl, 1% Nonidet P‐40, 3 mm EDTA, and a protease inhibitor mixture (Roche, Basel, Switzerland). Whole‐cell lysates (10 μg) were separated by sodium dodecyl sulfate–polyacrylamide gel electrophoresis, blotted onto polyvinylidene fluoride membranes (Millipore, Billerica, MA, USA), and blocked with 5% bovine serum albumin for 1 h at room temperature (22–25 °C). The membranes were then probed with the appropriate antibodies and antigen–antibody complexes were detected by enhanced chemiluminescence (GE Healthcare, Chicago, IL, USA).

### Enzyme‐linked immunosorbent assay (ELISA) for IL‐6

The amount of IL‐6 protein in 24‐h culture supernatants after LPS stimulation was measured at 450 nm using a plate reader (PerkinElmer, Waltham, MA, USA) and using the Mouse IL‐6 ELISA kit (R&D Systems, Minneapolis, MD, USA).

### Image and statistical analyses

Phase contrast microscopy images were taken using the Olympus IX73 inverted microscope. The macrophage surface area and signal of western blot bands were analyzed using Image J. All the assays were performed in triplicate. Statistical analyses were performed using the Student's *t*‐test or differences among more than two groups were analyzed using one‐way ANOVA followed by Tukey–Kramer's *post‐hoc* tests, using graphpad prism 9 (GraphPad Software, San Diego, CA, USA). The *P* values of < 0.05 and < 0.01 were considered statistically significant.

## Results

### Contribution of physiological stress to the inflammatory response

**Fig. 1:**
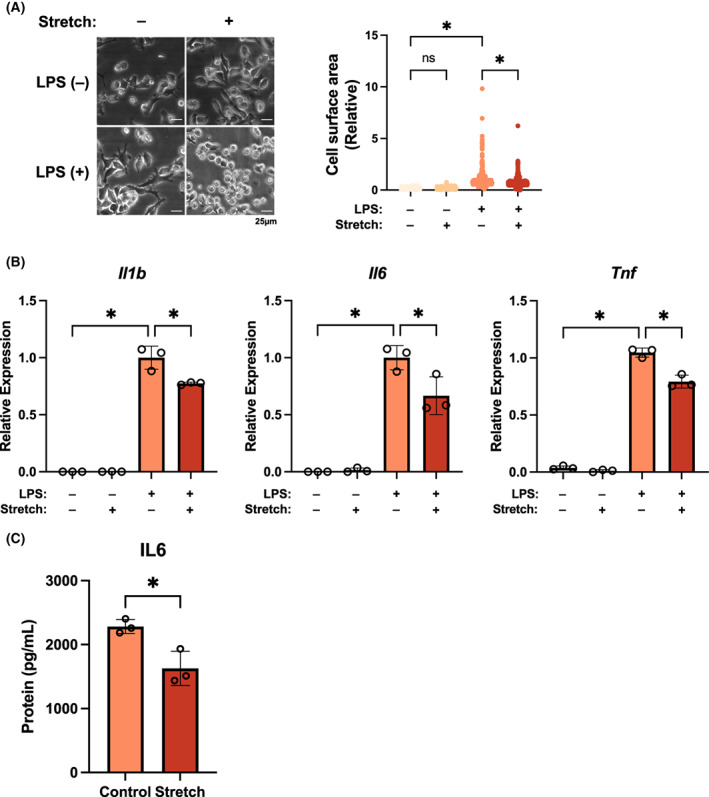
Involvement of physiological stress signaling in the innate immune response. (A) Morphological changes in Raw 264.7 cells after 4‐h lipopolysaccharide (LPS, 100 ng·mL^−1^) stimulation with or without 5% cyclic stretch stress. *n* = 307–374 in each group. (B) Quantitative reverse transcription‐polymerase chain reaction analysis of IL‐6, IL‐1β, and TNF mRNA expression after 4 h of LPS stimulation and (C) enzyme‐linked immunosorbent assay of IL‐6 after 24 h of LPS stimulation in Raw 264.7 cells treated with 5% cyclic stretch stress or without cyclic stretch stress. Comparisons between two groups were made using two‐tailed Student's *t*‐tests. Differences among more than two groups were analyzed using one‐way ANOVA followed by Tukey–Kramer's *post‐hoc* tests. Values of *P < 0.05 were considered statistically significant [mean and SD of triplicate (B, C)]. Scale bar = 25 μm.

To investigate the role of physiological stretch stress on the inflammatory response, we studied the inflammatory response induced by LPS stimulation under physiological cyclic stretch stress conditions. First, we determined whether physiological stretch stress affects the morphology of Raw264.7 mouse macrophage cells. Based on the results of quantitative surface area analysis, macrophage surface area decreased when physiological cyclic stretch was applied to Raw264.7 cells (Fig. 1A). The inflammatory response and cell area are closely related, with increased activation of the inflammatory response leading to increased cell area [11]. Therefore, we then examined the role of physiological stress on mRNA expression of inflammatory cytokines induced by LPS stimulation. Physiological stress suppressed the mRNA expression of inflammatory cytokines TNF, IL‐6, and IL‐1β and IL‐6 protein expression after stimulation with LPS (Fig. 1B,C). These data indicated that physiological stretch stress was intimately involved in the regulation of the inflammatory response during the activation of the innate immune response.

### Suppression of NF‐κB phosphorylation

**Fig. 2:**
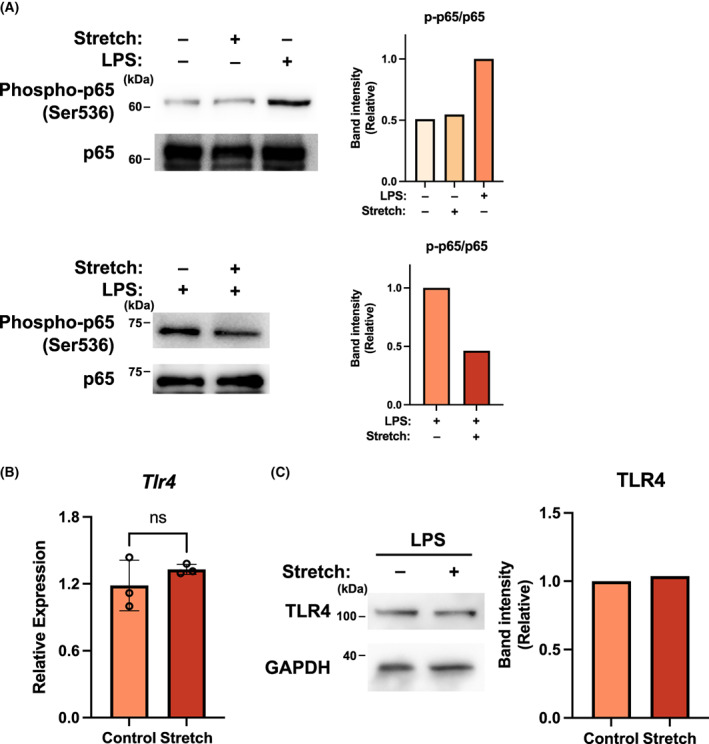
Cyclic stretch negatively regulates the phosphorylation of p65/NF‐kB at Ser536. (A) Immunoblot analysis of phosphorylation of p65/NF‐κB in Raw 264.7 macrophages treated with or without 5% cyclic stretch stress. Right, band intensity of p65 phosphorylation relative to p65 assessed by densitometry. (B) Quantitative reverse transcription‐polymerase chain reaction analysis of toll‐like receptor (TLR)‐4 mRNA expression and (C) TLR4 protein expression after lipopolysaccharide stimulation in Raw 264.7 macrophages treated with or without 5% cyclic stretch stress. Right, band intensity of TLR4 assessed by densitometry. NS indicates not significant, Student's two‐tailed *t*‐test [mean and SD of triplicate (B)].

Next, we examined the effect of physiological stretch stress on the TLR‐mediated induction of inflammatory cytokine expression. LPS stimulation of macrophages results in phosphorylation of NF‐κB, a transcription factor that is known to translocate into the nucleus, causing induction of pro‐inflammatory cytokines [12, 13] Physiological stretch stress significantly suppressed LPS‐induced inflammatory cytokine mRNA expression (Fig. 1B). Consistent with these results, NF‐κB phosphorylation at Ser536 in LPS‐stimulated cells was suppressed by stretch stress, whereas the amount of NF‐κB in the cells did not change markedly (Fig. 2A). Furthermore, no changes were observed in the mRNA expression of TLR4, which is known as the recognition receptor of LPS (Fig. 2B). We also examined the protein level of TLR4 by immunoblot analysis (Fig. 2C), which was not affected by physiological stretch stress. These results suggested that physiological stretch stress was a potent suppressor of the TLR‐mediated inflammatory response via the regulation of NF‐κB phosphorylation.

### Physiological stretch stress attenuates TBK1 expression

**Fig. 3:**
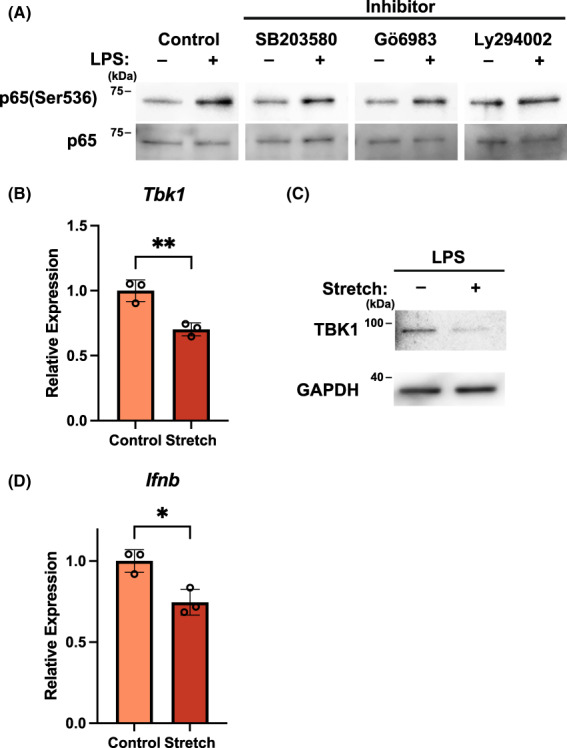
Physiological stress involves tank‐binding kinase (TBK)‐1 mRNA expression. (A) Phosphorylation of NF‐κB p65 in Raw 264.7 macrophages pretreated with inhibitors and then stimulated with lipopolysaccharide (LPS). (B) Quantitative analysis of TBK1 mRNA expression by qRT‐PCR and (C) TBK1 protein expression after LPS stimulation in Raw 264.7 macrophages treated with or without 5% cyclic stretch stress. (D) IFN‐β mRNA expression was measured by quantitative reverse transcription‐polymerase chain reaction under the same conditions. **P < 0.01, *P < 0.05, Student's two‐tailed *t*‐test [mean and SD of triplicate (B, D)].

We next investigated the mechanism of regulation of NF‐κB phosphorylation by physiological stretch stress. Different signaling pathways are known to be activated downstream of TLR4 [14]. Activation of TLR4 transmits signals to the adaptor protein Myd88, which leads to the activation of NF‐κB. TIR‐domain‐containing adapter‐inducing interferon‐β can also activate the NF‐κB pathway via TAK1. NF‐κB is known to be a key transcription factor in inflammatory signaling [4]. We therefore investigated whether NF‐κB phosphorylation levels were regulated by LPS treatment. We analyzed the phosphorylation at Ser536 in the NF‐κB pathway using three kinase inhibitors, revealing that phosphorylation of NF‐κB p65 was unaffected by these inhibitors, which indicated the presence of other important phosphorylation regulatory pathways (Fig. 3A). TBK1 is a member of the IkB kinase (IKK) family, which is reportedly involved in p65 phosphorylation [15, 16]. We then measured the mRNA expression of kinases involved in the TBK1 pathway by qRT‐PCR. Interestingly, TBK1 mRNA expression was significantly suppressed by physiological stretch stress (Fig. 3B), and its protein level was also suppressed (Fig. 3C). Further, IFN‐β mRNA expression was significantly suppressed in correlation with the suppression of TBK1 expression by physiological stretch stress (Fig. 3D). These findings suggested that physiological stretch stress suppressed TBK1 expression to negatively regulate the inflammatory response via suppression of NF‐κB p65 phosphorylation.

### Role of TBK1 in NF‐kB phosphorylation

**Fig. 4:**
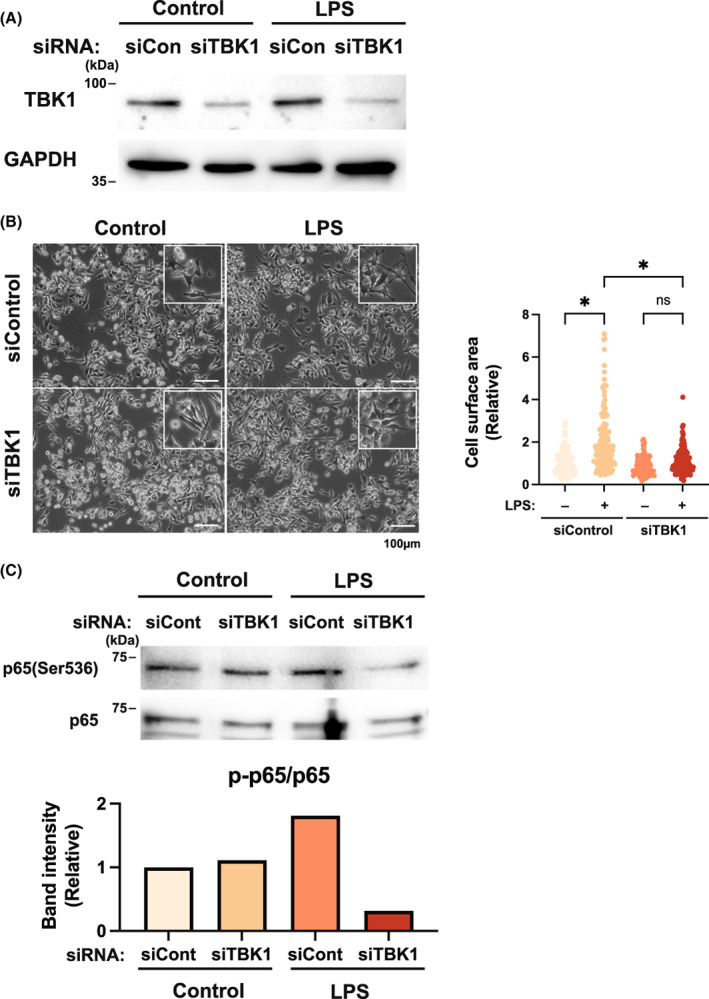
The function of tank‐binding kinase (TBK)‐1 in NF‐kB phosphorylation. (A) Immunoblot analysis of TBK1 expression in Raw 264.7 cells transfected with control siRNA (siCon) or with TBK1 siRNA (siTBK1). (B) Changes in cell morphology after treatment with lipopolysaccharide (LPS) for 4 h were suppressed by TBK1 siRNA. *n* = 145–193 in each group. (C) Immunoblot analysis of NF‐kB phosphorylation after LPS stimulation for 4 h in Raw 264.7 cells transfected with siRNA, siCon, or siTBK1. Differences among more than two groups were analyzed using one‐way ANOVA followed by Tukey–Kramer's *post‐hoc* tests. Values of *P < 0.05 were considered statistically significant (mean and SD). Scale bar = 100 μm.

To further evaluate the function of TBK1 in the TLR4‐mediated inflammatory response, we transfected macrophages with synthetic siRNA and analyzed the suppression of TBK1 protein by immunoblot analysis (Fig. 4A). We then examined the morphological changes in TBK1‐knockdown macrophages after LPS stimulation and found that cell elongation and spreading were inhibited (Fig. 4B). These results suggested that morphological changes in macrophages induced by LPS stimulation were suppressed by inhibiting the phosphorylation of NF‐κB p65 subunit for the induction of inflammatory cytokines. Therefore, we confirmed both the phosphorylation of the NF‐κB p65 subunit by immunoblotting and its suppression in a TBK1‐dependent manner (Fig. 4C). These results suggested that physiological stress played a crucial role in the innate immune response via the regulation of TBK1 expression.

## Discussion

Physiological stretch stress, such as movement, plays an important role in homeostasis [17]. Some studies have demonstrated that mechanical stretch directly regulates cell function and inflammatory response [3, 18, 19, 20]. Increased levels of pro‐inflammatory cytokines, including IL‐1β, IL‐6, IL‐8, and tumor necrosis factor‐alpha, have been reported in many cell types such as chondrocytes, osteoblasts, temporomandibular joint synovial cells, and human dental pulp cells receiving various types of stress [21]. Our preliminary observation also supported that LPS‐triggered expression of Il6 mRNA was induced in Raw264.7 cells treated with 20% mechanical stretch (unpublished data).

In this context, we speculated that weak physiological stress may act as positive or negative signaling in the TLR‐mediated induction of inflammatory cytokines.

Activation of TLR signaling has been reported to be important for the activation of innate immune responses [4]. In particular, TLR4 reportedly recognizes not only PAMPs but also endogenous DAMPs such as high mobility group box 1 protein, which are released when host cells are damaged, and is associated with inflammatory and autoimmune diseases [22]. However, the relationship between the activation of innate immune signaling and physiological stress has not been elucidated in detail. In this study, we found that physiological stretch stress regulated the inflammatory response via the regulation of TBK1 gene expression in macrophages.

LPS‐stimulated cell extension and inflammatory responses are known to be closely linked [11]. We found that morphological changes in LPS‐stimulated mouse macrophage Raw 264.7 cells were significantly suppressed by the application of 5% mechanical stretch. Consistent with these results, inflammatory cytokine mRNA expression was also suppressed by physiological stress. We further confirmed that LPS‐induced IL‐6 production was also significantly suppressed by physiological stress.

LPS stimulation leads to the activation of cytosolic protein kinases such as IKK and TBK1, which in turn activate transcription factors NF‐κB and IRF3 or IRF7, respectively [5]. TLR4‐mediated activation after ligand binding occurs via the regulation of a multistep process that includes the phosphorylation of NF‐κB p65 subunit [15]. Chen et al. [23] demonstrated that acetylation of RelA(p65) at lysine 310 is regulated by prior phosphorylation of Ser536. Our data indicated that the physiological stretch signal was associated with the TLR4 pathway after LPS stimulation and suppressed p65 phosphorylation at Ser536; however, did not affect TLR4 mRNA expression and protein level. We found no change in the expression of weakly phosphorylated p65 and p65 when only cyclic stretch was analyzed, suggesting that cyclic stretch, which occurs simultaneously with ligand, may be important for this phosphorylation.

The TLR4 downstream pathway is known to involve kinases such as p38MAPK, PKC, and PI3K [4]. Our data indicated that these enzymes were not related to the phosphorylation of p65 at Ser536. Christian et al. reported several kinases that phosphorylate Ser536, including IKKα, ribosomal subunit S6 kinase 1, IKKβ, IKKε, and NF‐κB activating kinase/TBK1 [13]. Our data indicated that TBK1 mRNA expression and protein levels were suppressed by physiological stretch stress. Signals from the extracellular matrix (ECM) involving mechanotransduction have been reported to be a fundamental cellular input that maintains cell proliferation, opposes cell death, and regulates differentiation [24]. Recently, the Hippo‐YAP pathway has been reported to be involved in the regulation of TBK1‐mediated innate immune responses. It has also become clear that innate immune signaling is involved in the regulation of the Hippo‐YAP pathway [25]. Although it is becoming clear that the Hippo‐YAP pathway involving mechanotransduction is closely linked to the immune system, the regulatory mechanism of TBK1 mRNA expression requires further investigation. Consistently, TBK1‐regulated interferon beta mRNA, which is a type I interferon, was also considerably suppressed by physiological mechanical stress. In addition, our results supported that suppression of TBK1 by the synthetic siRNA suppressed the morphological changes and phosphorylation of NF‐κB p65 subunit at Ser536. The adaptor protein MAVS activates the downstream protein kinase TBK1, which then phosphorylates the transcription factor interferon regulatory factor 3 (IRF3), which drives type I IFN production [26]. Overexpression of TBK1 may restore these signals, further suggesting that TBK1‐mediated ligand stimulation is affected by gene expression by cyclic stretch.

Together, these findings suggest that physiological mechanical stress regulates the suppression of TBK1 gene expression, presumably by transcriptional regulation. However, other mechanisms may be operative at the same time, since a portion of the activated enzyme may be regulated into cells to exert its activities. Our findings support the importance of physiological movement in the innate immune response by elucidating the molecular mechanisms underlying the effect of cyclic stretching on macrophages.

## Conflict of interest

The authors declare no conflict of interest.

## Author contributions

AN and SH constructed the concept of the whole study; AN, SH, AH, RY and YC performed the investigation; SH wrote the original manuscript; SH, RO, and YO edited and revised the manuscript; and YO and RO supervised the project and funding support. All authors have read the manuscript and approved for publication.

## Data Availability

The data that support the findings of this study are available in the figures of the published article.

## References

[1] Lavin Y , Winter D , Blecher‐Gonen R , David E , Keren‐Shaul H , Merad M , et al. Tissue‐resident macrophage enhancer landscapes are shaped by the local microenvironment. Cell. 2014;159:1312–26. 25480296 10.1016/j.cell.2014.11.018 PMC4437213

[2] Gillespie PG , Walker RG . Molecular basis of mechanosensory transduction. Nature. 2001;413:194–202. 11557988 10.1038/35093011

[3] Maruyama K , Nemoto E , Yamada S . Mechanical regulation of macrophage function – cyclic tensile force inhibits NLRP3 inflammasome‐dependent IL‐1β secretion in murine macrophages. Inflamm Regen. 2019;39:3. 30774738 10.1186/s41232-019-0092-2 PMC6367847

[4] Kawai T , Akira S . The role of pattern‐recognition receptors in innate immunity: update on toll‐like receptors. Nat Immunol. 2010;11:373–84. 20404851 10.1038/ni.1863

[5] Kawai T , Akira S . Toll‐like receptors and their crosstalk with other innate receptors in infection and immunity. Immunity. 2011;34:637–50. 21616434 10.1016/j.immuni.2011.05.006

[6] Lim C‐G , Jang J , Kim C . Cellular machinery for sensing mechanical force. BMB Rep. 2018;51:623–9. 30293551 10.5483/BMBRep.2018.51.12.237 PMC6330935

[7] Katsumi A , Orr AW , Tzima E , Schwartz MA . Integrins in mechanotransduction. J Biol Chem. 2004;279:12001–4. 14960578 10.1074/jbc.R300038200

[8] Iqbal J , Zaidi M . Molecular regulation of mechanotransduction. Biochem Biophys Res Commun. 2005;328:751–5. 15694410 10.1016/j.bbrc.2004.12.087

[9] Gao X , Wei T , Liao B , Ai J , Zhou L , Gong L , et al. Physiological stretch induced proliferation of human urothelial cells via integrin alpha6‐FAK signaling pathway. NeurourolUrodyn. 2018;37:2114–20. 10.1002/nau.23572 29953644

[10] Freese C , Schreiner D , Anspach L , Bantz C , Maskos M , Unger RE , et al. In vitro investigation of silica nanoparticle uptake into human endothelial cells under physiological cyclic stretch. Part Fibre Toxicol. 2014;11:68. 25539809 10.1186/s12989-014-0068-y PMC4318365

[11] Lee H‐S , Stachelek SJ , Tomczyk N , Finley MJ , Composto RJ , Eckmann DM . Correlating macrophage morphology and cytokine production resulting from biomaterial contact. J Biomed Mater Res A. 2013;101:203–12. 22847892 10.1002/jbm.a.34309 PMC3488130

[12] Noursadeghi M , Tsang J , Haustein T , Miller RF , Chain BM , Katz DR . Quantitative imaging assay for NF‐kappaB nuclear translocation in primary human macrophages. J Immunol Methods. 2008;329:194–200. 18036607 10.1016/j.jim.2007.10.015 PMC2225449

[13] Christian F , Smith EL , Carmody RJ . The regulation of NF‐κB subunits by phosphorylation. Cell. 2016;5:12. 10.3390/cells5010012 PMC4810097 26999213

[14] Kawai T , Akira S . TLR signaling. Cell Death Differ. 2006;13:816–25. 16410796 10.1038/sj.cdd.4401850

[15] Buss H , Dörrie A , Schmitz ML , Hoffmann E , Resch K , Kracht M . Constitutive and interleukin‐1‐inducible phosphorylation of p65 NF‐{kappa}B at serine 536 is mediated by multiple protein kinases including I{kappa}B kinase (IKK)‐{alpha}, IKK{beta}, IKK{epsilon}, TRAF family member‐associated (TANK)‐binding kinase 1 (TBK). J Biol Chem. 2004;279:55633–43. 15489227 10.1074/jbc.M409825200

[16] Sakurai H , Chiba H , Miyoshi H , Sugita T , Toriumi W . IkappaB kinases phosphorylate NF‐kappaB p65 subunit on serine 536 in the transactivation domain. J Biol Chem. 1999;274:30353–6. 10521409 10.1074/jbc.274.43.30353

[17] Chapman GB , Durante W , Hellums JD , Schafer AI . Physiological cyclic stretch causes cell cycle arrest in cultured vascular smooth muscle cells. Am J Physiol Heart Circ Physiol. 2000;278:H748–54. 10710342 10.1152/ajpheart.2000.278.3.H748

[18] Carnevale D , Wenzel P . Mechanical stretch on endothelial cells interconnects innate and adaptive immune response in hypertension. Cardiovasc Res. 2018;114:1432–4. 29912294 10.1093/cvr/cvy148

[19] Chu S‐Y , Chou C‐H , Huang H‐D , Yen M‐H , Hong H‐C , Chao P‐H , et al. Mechanical stretch induces hair regeneration through the alternative activation of macrophages. Nat Commun. 2019;10:1524. 30944305 10.1038/s41467-019-09402-8 PMC6447615

[20] Gruber EJ , Leifer CA . Molecular regulation of TLR signaling in health and disease: mechano‐regulation of macrophages and TLR signaling. Innate Immun. 2020;26:15–25. 31955624 10.1177/1753425919838322 PMC6974875

[21] Kanjanamekanant K , Luckprom P , Pavasant P . Mechanical stress‐induced interleukin‐1beta expression through adenosine triphosphate/P2X7 receptor activation in human periodontal ligament cells. J Periodontal Res. 2013;48:169–76. 22881405 10.1111/j.1600-0765.2012.01517.x

[22] Gong T , Liu L , Jiang W , Zhou R . DAMP‐sensing receptors in sterile inflammation and inflammatory diseases. Nat Rev Immunol. 2020;20:95–112. 31558839 10.1038/s41577-019-0215-7

[23] Chen L‐F , Williams SA , Mu Y , Nakano H , Duerr JM , Buckbinder L , et al. NF‐kappaB RelA phosphorylation regulates RelA acetylation. Mol Cell Biol. 2005;25:7966–75. 16135789 10.1128/MCB.25.18.7966-7975.2005 PMC1234328

[24] Dupont S . Role of YAP/TAZ in cell‐matrix adhesion‐mediated signalling and mechanotransduction. Exp Cell Res. 2016;343:42–53. 26524510 10.1016/j.yexcr.2015.10.034

[25] Wang S , Zhou L , Ling L , Meng X , Chu F , Zhang S , et al. The crosstalk between hippo‐YAP pathway and innate immunity. Front Immunol. 2020;11:323. 32174922 10.3389/fimmu.2020.00323 PMC7056731

[26] Hagan RS , Torres‐Castillo J , Doerschuk CM . Myeloid TBK1 signaling contributes to the immune response to influenza. Am J Respir Cell Mol Biol. 2019;60:335–45. 30290124 10.1165/rcmb.2018-0122OC PMC6397979

